# Accurate classification of benign and malignant breast tumors in ultrasound imaging with an enhanced deep learning model

**DOI:** 10.3389/fbioe.2025.1526260

**Published:** 2025-06-25

**Authors:** Baoqin Liu, Shouyao Liu, Zijian Cao, Junning Zhang, Xiaoqi Pu, Junjie Yu

**Affiliations:** ^1^ Department of TCM gynecology, China-Japan Friendship Hospital, Beijing, China; ^2^ Department of TCM surgery, China-Japan Friendship Hospital, Beijing, China; ^3^ School of Biomedical Engineering, Tsinghua Medicine, Tsinghua University, Beijing, China; ^4^ Graduate School, Beijing University of Chinese Medicine, Beijing, China; ^5^ Department of Diagnostic Radiology, China-Japan Friendship Hospital, Beijing, China

**Keywords:** breast ultrasound, deep learning, deep separable convolution, attention mechanism, benign and malignant diagnosis

## Abstract

**Background:**

Breast cancer is the most common malignant tumor in women worldwide, and early detection is crucial to improving patient prognosis. However, traditional ultrasound examinations rely heavily on physician judgment, and diagnostic results are easily influenced by individual experience, leading to frequent misdiagnosis or missed diagnosis. Therefore, there is a pressing need for an automated, highly accurate diagnostic method to support the detection and classification of breast cancer. This study aims to build a reliable breast ultrasound image benign and malignant classification model through deep learning technology to improve the accuracy and consistency of diagnosis.

**Methods:**

This study proposed an innovative deep learning model RcdNet. RcdNet combines deep separable convolution and Convolutional Block Attention Module (CBAM) attention modules to enhance the ability to identify key lesion areas in ultrasound images. The model was internally validated and externally independently tested, and compared with commonly used models such as ResNet, MobileNet, RegNet, ViT and ResNeXt to verify its performance advantage in benign and malignant classification tasks. In addition, the model’s attention area was analyzed by heat map visualization to evaluate its clinical interpretability.

**Results:**

The experimental results show that RcdNet outperforms other mainstream deep learning models, including ResNet, MobileNet, and ResNeXt, across all key evaluation metrics. On the external test set, RcdNet achieved an accuracy of 0.9351, a precision of 0.9168, a recall of 0.9495, and an F1-score of 0.9290, demonstrating superior classification performance and strong generalization ability. Furthermore, heat map visualizations confirm that RcdNet accurately attends to clinically relevant features such as tumor edges and irregular structures, aligning well with radiologists’ diagnostic focus and enhancing the interpretability and credibility of the model in clinical applications.

**Conclusion:**

The RcdNet model proposed in this study performs well in the classification of benign and malignant breast ultrasound images, with high classification accuracy, strong generalization ability and good interpretability. RcdNet can be used as an auxiliary diagnostic tool to help physicians quickly and accurately screen breast cancer, improve the consistency and reliability of diagnosis, and provide strong support for early detection and precise diagnosis and treatment of breast cancer. Future work will focus on integrating RcdNet into real-time ultrasound diagnostic systems and exploring its potential in multi-modal imaging workflows.

## 1 Introduction

Breast cancer stands as one of the most common malignancies among women worldwide, representing a major public health challenge due to its high incidence and mortality rates. In 2020 alone, breast cancer accounted for approximately 2.3 million new cases and around 685,000 deaths globally, underscoring its significant impact on both individual lives and healthcare systems (Beck et al., 2014). Annually, breast cancer causes hundreds of thousands of deaths among women, with the associated economic burden being considerable. This economic impact stems not only from direct medical costs but also from indirect costs, including lost productivity and long-term care expenses (Sung et al., 2021; Bray et al., 2020).

Traditional diagnostic methods, such as mammography, ultrasound, and magnetic resonance imaging (MRI), are crucial but present limitations, especially in early disease detection. These techniques are prone to false negatives and often miss smaller or less aggressive tumors, creating a need for more accurate and sensitive diagnostic methods (Lane et al., 2014; Houssami et al., 2011; Elder et al., 2023).

Advances in machine learning (ML) provide promising pathways to enhance breast cancer diagnostic accuracy, particularly through the analysis of ultrasound images (Zhang et al., 2023). By integrating techniques such as support vector machines and principal component analysis, studies have reported improved classification accuracy, with some achieving rates as high as 89%, thereby opening new avenues for early detection (Liu et al., 2023).

Previous research has demonstrated that ML algorithms can effectively analyze complex imaging data, improving detection rates by identifying subtle patterns that may be overlooked by human observers (Budillon and Schirinzi, 2015). For example, ML models have shown significant potential in differentiating between benign and malignant masses based on ultrasound characteristics, paving the way for earlier and more accurate diagnoses (Wang et al., 2022). This growing recognition of the need for innovative diagnostic tools underscores the importance of complementary methods that can provide more reliable assessments of breast abnormalities (Ponso et al., 2022).

This study investigates the application of ML techniques for breast ultrasound image analysis, aiming to develop a robust model to enhance the detection and characterization of breast lesions. Utilizing a diverse ultrasound image dataset, we will employ various ML algorithms to assess their effectiveness in distinguishing between malignant and benign conditions. Through the capabilities of ML, this study seeks to contribute to the current knowledge base and address critical challenges in the early detection of breast cancer. Ultimately, the integration of ML into breast cancer diagnostics has the potential not only to improve clinical outcomes but also to reduce the emotional and financial toll associated with this prevalent disease (Economopoulou et al., 2020).

**FIGURE 1:**
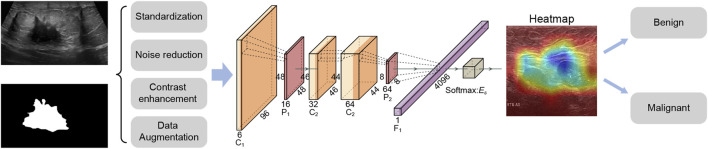
Workflow diagram of this study.

Specifically, this study used a deep learning strategy to develop a benign and malignant classification model for breast cancer ultrasound images, aiming to provide doctors with accurate auxiliary diagnostic tools. The data was preprocessed by cleaning and standardization, and combined with data enhancement techniques such as random cropping and horizontal flipping to improve the robustness of the model. In the model part, multiple machine learning and deep learning models were constructed for comparison. The RcdNet model proposed in the end improved the EfficientNet architecture and incorporated innovative designs such as deep separable convolution, CBAM module and pre-trained weights to improve classification performance. The model outperformed other comparison models in multiple indicators, verifying its superiority in the task of breast cancer ultrasound image classification. The workflow of this study is shown in Figure 1.

## 2 Materials and methods

### 2.1 Data collection

The data of this study mainly comes from two different datasets to ensure the diversity of data and the generalization ability of the model. First, the data of the training set and the validation set are selected from the public dataset on the Kaggle platform (https://www.kaggle.com/datasets/aryashah2k/breast-ultrasound-images-dataset), which contains 780 breast ultrasound images divided into three categories: normal, benign, and malignant (133 normal (17.1%), 437 benign (56.0%), and 210 malignant (26.9%)). The images are uniform in size, with a resolution of 500 × 500 pixels and saved in PNG format. This dataset provides detailed annotation information of the images and is suitable for the classification and segmentation tasks of breast ultrasound images. Through this grouping method, the diversity of the training set and validation set images can be ensured to avoid overfitting.

To evaluate the performance of the model, we used another independent dataset as the test set. The data came from the “Breast Ultrasound Dataset B” provided by Manchester Metropolitan University (Yap et al., 2018). This dataset contains 163 breast ultrasound images with a resolution of 500 × 500 pixels, stored in PNG format, of which 53 are malignant tumor images and 110 are benign tumor images. Each image has clear annotation information. The image types and annotation quality of this dataset are high, which is suitable for independent testing and validation of the model. The advantage of using this dataset as a test set is that it can provide additional diversity and data independence, which helps us evaluate the generalization performance of the model more objectively. To ensure that there are no duplicate images and independence between datasets, we strictly screened and controlled the training, validation, and test sets to minimize the possible bias in cross-validation. At the same time, to further ensure the balance of the dataset, we ensured that the ratio of benign and malignant samples was balanced in both training and test data, thereby improving the model’s ability to recognize samples of different categories.

### 2.2 Data labeling and preprocessing

In terms of data annotation, all breast ultrasound images were reviewed and annotated by professional physicians to ensure the accuracy of the data (Yap et al., 2018). In the data preprocessing stage, in order to enhance the expressiveness of the model and reduce the noise caused by differences in image quality, we standardized all images. First, the size of all images was adjusted to a uniform 224 × 224 pixels to ensure input consistency and reduce the amount of calculation. Then, the images were contrast enhanced and denoised to improve image clarity so that the model can better capture the characteristics of the lesion area. In addition, we applied data augmentation techniques such as random cropping and horizontal flipping to increase the diversity of samples and help the model learn more image transformation features, thereby improving its robustness and generalization ability. In the data normalization process, we used standard normalization parameters, that is, normalizing the pixel values of the image to a range of a mean of 0.485 and a standard deviation of 0.229. This process helps to reduce the interference caused by different image brightness and contrast, allowing the model to focus more on important pathological features. In addition, data augmentation techniques also include random rotation and micro-scaling to further expand sample diversity, reduce the model’s dependence on specific image features, and thereby improve adaptability to unseen data.

### 2.3 Deep learning model construction

#### 2.3.1 RcdNet design

RcdNet model is designed based on EfficientNet. Through multi-layer optimization and innovative modules of deep learning, it achieves high accuracy and robustness for the benign and malignant classification task of breast ultrasound images. The name of RcdNet comes from the combination of its core design ideas and key modules. The name “Rcd” stands for “Resilient Convolutional and Deep Network,” highlighting the three main characteristics of the model.

RcdNet incorporates an advanced Depthwise Separable Convolution (DSC) module to improve feature extraction efficiency while significantly reducing computational complexity (Zhang et al., 2024). Unlike traditional convolution operations, which simultaneously learn spatial and channel correlations at a high computational cost, the DSC mechanism decomposes this process into two distinct stages. First, depthwise convolution applies a spatial filter to each input channel independently, effectively capturing intra-channel spatial patterns. Then, pointwise convolution (a 1 × 1 convolution) is used to integrate the features across channels. This structural design dramatically reduces the number of parameters and the amount of computation required, enabling the model to maintain high performance even when applied to large-scale, high-resolution ultrasound images. In clinical contexts where computational resources may be limited, such as in portable or real-time diagnostic systems, this lightweight structure greatly enhances the practicality and scalability of the RcdNet model.

In addition to its efficient convolutional backbone, RcdNet integrates a Convolutional Block Attention Module (CBAM) to enhance the model’s ability to focus on diagnostically critical regions within the breast ultrasound images (Omega Boro and Nandi, 2025). CBAM sequentially applies channel attention and spatial attention mechanisms to refine the intermediate feature maps. The channel attention mechanism emphasizes feature channels that are more informative for the classification task, thereby strengthening the model’s ability to distinguish subtle lesion differences. Subsequently, the spatial attention module assigns higher weights to regions of interest within each feature map, guiding the network to concentrate on specific tumor boundaries or structural irregularities commonly associated with malignancy. By combining these two attention mechanisms, CBAM enables RcdNet to suppress redundant or noisy background information and prioritize key anatomical features, ultimately improving the interpretability and accuracy of the classification outcomes.

In the model initialization stage, RcdNet uses pre-trained weights. These pre-trained weights have learned certain common features on large-scale datasets, so they can accelerate the convergence of the model during fine-tuning. The introduction of pre-trained weights not only reduces the model’s dependence on training data, but also effectively reduces the training time, allowing RcdNet to achieve better performance on limited breast ultrasound image data. In order to improve the generalization ability of the model, RcdNet applies label smoothing technology in the cross entropy loss function. Label smoothing can prevent the model from being overconfident and avoids excessive predictions for a certain category, which is particularly important for breast cancer classification tasks. In terms of data enhancement, RcdNet introduces Mixup technology, which is to linearly combine two samples and their labels in a certain proportion to generate new training samples. This data enhancement method increases the diversity of data, further improves the robustness of the model, and effectively reduces the occurrence of overfitting. These optimizations make RcdNet a reliable and practical auxiliary tool in breast cancer diagnosis.

#### 2.3.2 Other deep learning models

In order to fully verify the performance of RcdNet in the classification of benign and malignant breast ultrasound images, we selected several representative deep learning models for comparison, including ResNet, MobileNet, RegNet, ViT and ResNeXt. These models have their own characteristics in different architectures and optimization strategies, and can provide multi-angle performance evaluation.

ResNet (Residual Network) has a stable performance in image classification due to its residual connection structure and is particularly suitable for extracting deep features. We chose ResNet50 for comparison, mainly because it can maintain gradient stability in deep networks, making the model more capable of learning complex features and thus better identifying subtle differences in breast images (Pour et al., 2024). MobileNet is a lightweight model designed specifically for mobile devices and low computing resource environments. We chose MobileNetV2 to evaluate the performance of models with high computational efficiency and low resource requirements in breast ultrasound image classification tasks, especially the ability to reduce computational burden while maintaining model accuracy (Huang et al., 2022). RegNet is a new type of convolutional network that optimizes its structure through automated design to adapt to datasets of different sizes (Yin et al., 2023). We chose RegNet because of its excellent performance in parameter efficiency and its ability to provide robust classification results while reducing the number of parameters, which is particularly important for medical image classification tasks.

ViT (Vision Transformer) introduces a self-attention mechanism that can capture global features and is suitable for processing complex images. We chose ViT to evaluate the performance of Transformer-based models in image classification, especially for understanding the global information of ultrasound images, although its performance on small-scale datasets may not be as good as that of convolutional networks (Umamaheswari and Babu, 2024). ResNeXt combines the advantages of grouped convolution and residual connections, making the model more flexible in capturing local features. ResNeXt was chosen because it optimizes computational efficiency while maintaining accuracy and is suitable for tasks that require fine classification, such as distinguishing benign from malignant breast ultrasound images (Sepahvand and Abdali-Mohammadi, 2023).

### 2.4 Experimental setup

We have configured the training and validation process in detail. The training dataset is divided into a training set and an internal validation set in a ratio of 7:3 to ensure that the model has sufficient sample size during training and validation, and then the generalization of the model is verified again on the external test set. The hardware device used for model training is the NVIDIA RTX 3090 graphics card. The experimental environment is based on the Ubuntu 20.04 operating system. The deep learning framework selected is PyTorch 1.9.0, which supports CUDA 11.2 to make full use of GPU acceleration. In the process of data processing and image enhancement, OpenCV and Pillow libraries were used. The batch size during training was set to 32, the initial value of the learning rate was 0.001, and the cosine annealing scheduling strategy was used to gradually adjust the learning rate.

During the model optimization process, we selected the Adam optimizer with an initial learning rate of 0.001 and a weight decay coefficient of 1e-4. In addition, regularization was introduced to further reduce overfitting. The hyperparameters in the experiment were fixed after preliminary tuning, so that the experimental process could be reproduced and stable training results could be guaranteed. The entire training process ran for 30 epochs, and after the end, all models converged without overfitting.

### 2.5 Model evaluation

During the model evaluation phase, we used a series of evaluation indicators to comprehensively measure the performance of the model in the task of classifying benign and malignant breast ultrasound images. The main evaluation indicators include accuracy, precision, recall, F1-score, and the area under the receiver operating characteristic curve (AUC). These indicators can reflect the classification effect of the model from different angles (Powers, 2020). Accuracy represents the correctness of the overall classification, precision and recall respectively measure the model’s prediction accuracy and coverage of malignant samples, F1-score balances the relationship between precision and recall, and AUC shows the comprehensive performance of the model at different thresholds. The closer the value is to 1, the stronger the model’s discrimination ability.

## 3 Results

### 3.1 Internal validation results

**FIGURE 2:**
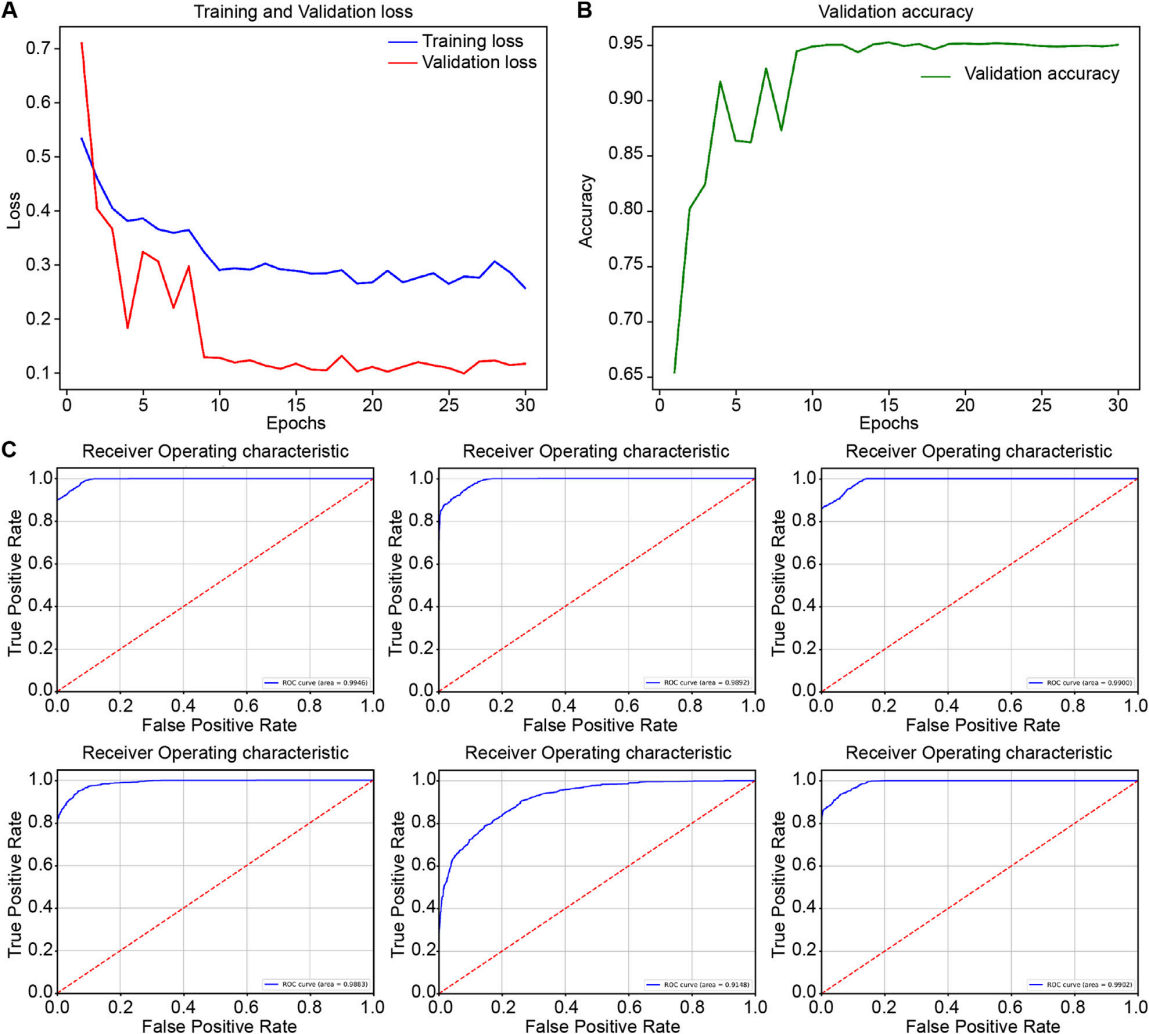
Results of model training and internal validation. **(A, B)** are the loss changes and accuracy changes of RcdNet training, respectively; **(C)** is the ROC curve of internal validation of each model, from top to bottom and from left to right, they are RcdNet, ResNet, MobileNet, RegNet, ViT and ResNeXt.

During the internal validation process, RcdNet demonstrated excellent classification performance. By plotting the training loss and accuracy curves of RcdNet, we observed that the model converged quickly during the training process, and the accuracy on the validation set gradually increased and finally stabilized above 0.95 (Figures 2A, B). At the same time, the ROC curve of RcdNet showed superiority, and its AUC value was close to 1, which was higher than other models (Figure 2C).

In addition, its main evaluation indicators are better than other comparison models, and the specific results are shown in Table 1. Specifically, RcdNet achieved the highest values in accuracy, precision, recall and F1-score, which are 0.9504, 0.9515, 0.9508 and 0.9504 respectively. These indicators show that RcdNet can not only identify malignant breast tumors with high accuracy, but also balance the accuracy and recall ability well, showing strong generalization. In the comparative experiment, although the performance of traditional models such as ResNet and MobileNet is relatively excellent, they are slightly lower than RcdNet in accuracy and F1-score, which are 0.9317 and 0.9306 respectively. The performance of the ViT model is slightly worse, especially in F1-score and accuracy, which are 0.8176 and 0.8178. This shows that RcdNet has higher classification ability and stronger robustness when dealing with complex ultrasound image classification tasks.

**TABLE 1:** Performance of each model in the internal validation set.

Method	Precision	Recall	F1-score	Accuracy
ResNet	0.9317	0.9314	0.9311	0.9311
MobileNet	0.9372	0.9316	0.9306	0.9308
RegNet	0.9393	0.9393	0.9391	0.9392
ViT	0.8203	0.8183	0.8176	0.8178
ResNeXt	0.9339	0.9393	0.9391	0.9337
RcdNet	0.9515	0.9508	0.9504	0.9504

### 3.2 External test results

On the external test set, RcdNet once again demonstrated excellent classification performance, with improvements in all indicators compared to other models (as shown in Table 2). In terms of precision, RcdNet reached 0.9168, slightly higher than the better-performing ResNeXt (0.9101), indicating that RcdNet is more accurate in predicting malignant cases. Similarly, RcdNet achieved a high score of 0.9495 in recall, which is better than all other comparison models, indicating that it performs better in detecting the coverage of malignant cases. In addition, RcdNet’s F1-score is 0.9290, indicating that its ability to balance precision and recall makes the overall classification effect of the model more robust. In terms of accuracy, RcdNet also leads the way, reaching 0.9351, which is an improvement over other models. Compared with traditional convolutional neural network models such as ResNet and MobileNet, RcdNet better captures the key features in breast ultrasound images through innovative deep separable convolution and attention modules, achieving higher classification accuracy.

**TABLE 2:** Performance of each model in the external test set.

Method	Precision	Recall	F1-score	Accuracy
ResNet	0.8901	0.9313	0.9020	0.9088
MobileNet	0.8960	0.9358	0.9084	0.9150
RegNet	0.8759	0.9198	0.8864	0.8934
ViT	0.8619	0.8957	0.8728	0.8825
ResNeXt	0.9101	0.9461	0.9227	0.9289
RcdNet	0.9168	0.9495	0.9290	0.9351

**FIGURE 3:**
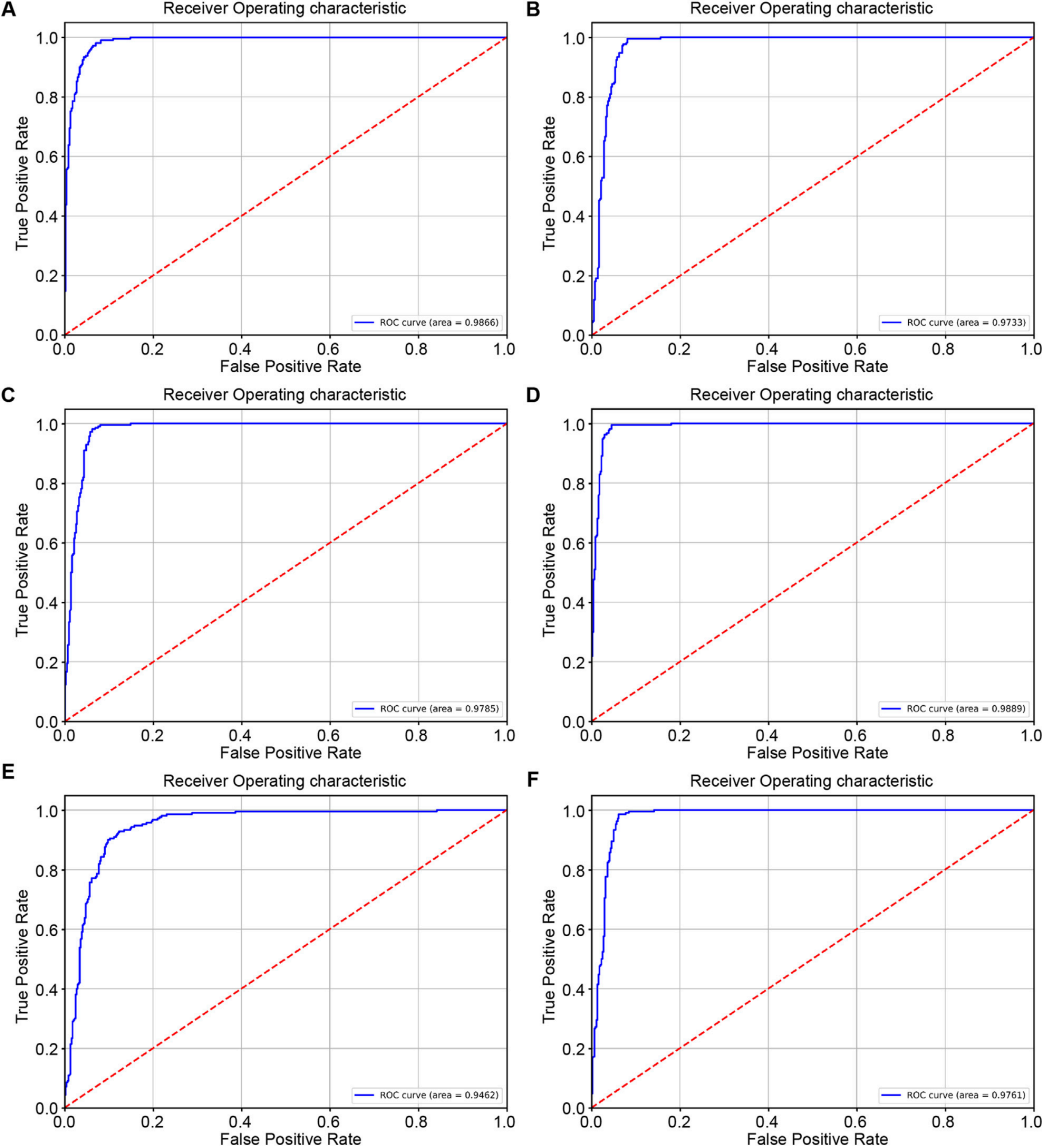
Results of external model testing. **(A–F)** represent the ROC curves and AUC values of RcdNet, ResNet, MobileNet, RegNet, ViT and ResNeXt, respectively.

The ROC curve shows the generalization performance of RcdNet, with an AUC of 0.9866, which is better than other mainstream models. This shows that under different decision thresholds, the discrimination ability of RcdNet always remains at a high level (Figure 3).

### 3.3 Model visualization and key area interpretation

To elucidate the interpretability of RcdNet and verify whether its classification decisions are grounded in clinically meaningful features, we conducted a detailed heat map analysis using Gradient-weighted Class Activation Mapping (Grad-CAM). This approach enables the visualization of the model’s attention distribution across the input image by highlighting regions that most strongly influence its predictions. By superimposing the heat maps on the original ultrasound images, we are able to observe which image features the model prioritizes when distinguishing between benign and malignant lesions.

**FIGURE 4:**
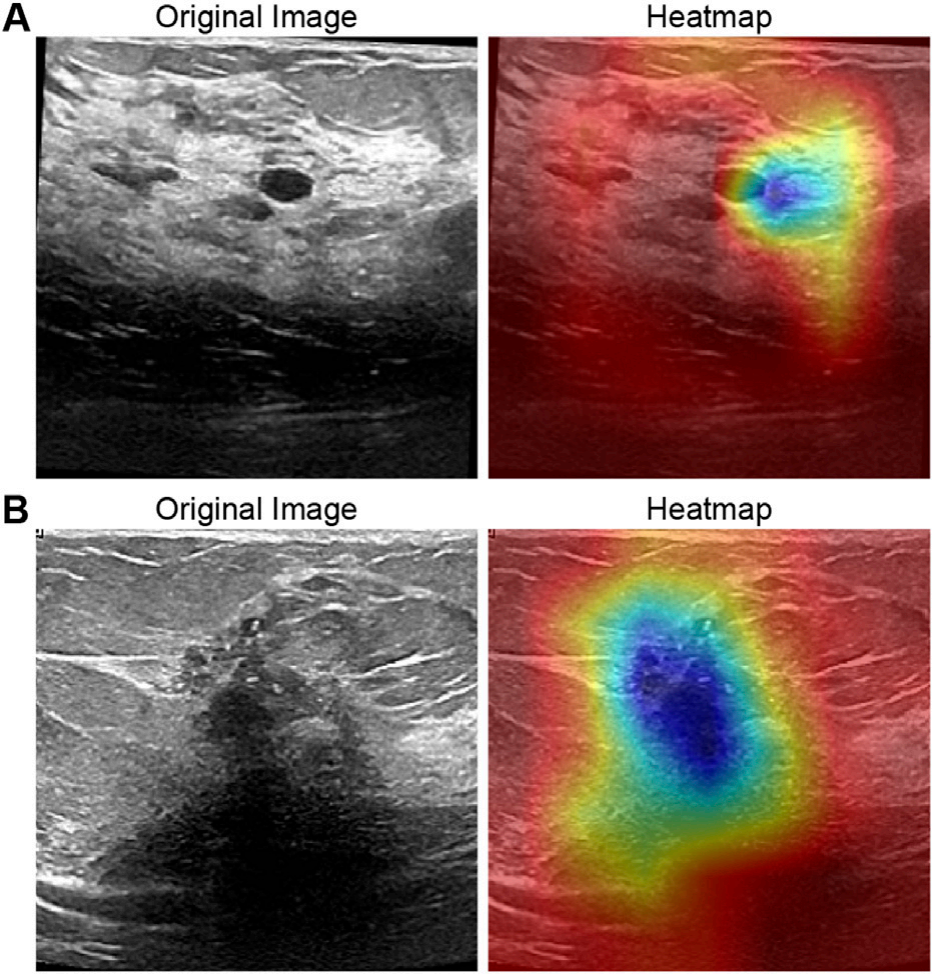
Grad-CAM activation heat map visualization of the model. **(A)** is a benign example and **(B)** is a malignant example.

In the benign lesion sample (Figure 4A), the heat map demonstrates that RcdNet concentrates on well-defined tumor boundaries and uniformly distributed internal echo regions—features that are characteristic of benign masses in clinical breast ultrasound. These lesions typically exhibit smooth contours, homogeneous echotexture, and limited disruption to surrounding tissue. The model’s attention pattern suggests a learned focus on these benign indicators, supporting the notion that RcdNet internalizes features analogous to those used by expert radiologists during diagnosis.

In contrast, the heat map for the malignant lesion (Figure 4B) reveals that RcdNet directs its attention to irregular tumor margins, burr-like extensions, and hypoechoic core regions—hallmarks of malignant behavior. These features often reflect invasive growth, poorly defined borders, and heterogeneity within the lesion, all of which are critical clinical indicators of malignancy. The model’s ability to accurately localize these regions demonstrates its capacity to extract and emphasize relevant pathological cues.

Overall, the comparison between benign and malignant cases indicates that RcdNet does not rely on spurious correlations or background artifacts, but rather focuses on anatomically and diagnostically significant regions. This level of interpretability enhances clinical trust in the model’s outputs and suggests strong potential for integration into diagnostic workflows as a reliable, explainable decision support system.

## 4 Discussion

This study proposed a deep learning-based breast ultrasound image benign and malignant classification model, RcdNet, and verified its effectiveness in breast cancer auxiliary diagnosis through a large number of experiments. Compared with the traditional convolutional neural network model, RcdNet has improved the classification accuracy and generalization ability of the model to a certain extent by introducing multiple optimizations such as deep separable convolution, CBAM attention module and pre-trained weights. In internal validation and external testing, RcdNet outperformed other comparison models in key indicators such as accuracy, precision, recall and F1-score, especially in complex breast ultrasound image classification tasks. This study also used heat map visualization technology to intuitively display the decision-making process of the model and revealed the focus areas of RcdNet in the identification of benign and malignant lesions. Through this interpretable analysis, we observed that the feature areas that the model focused on were basically consistent with the clinical diagnosis points for benign and malignant breast lesions, further verifying the reliability of RcdNet.

RcdNet performs well in the task of benign and malignant classification of breast ultrasound images, mainly due to its innovative architecture design and multiple optimization strategies. The incorporation of the deep separable convolution module effectively reduces the number of parameters and computational complexity of the model, so that the model still has strong feature extraction capabilities while maintaining high computational efficiency. This is particularly important for processing high-resolution ultrasound images, which can help the model focus on the subtle features of the lesion area, thereby improving the accuracy of classification. Secondly, the addition of the convolutional block attention module (CBAM) enhances the model’s ability in feature selection. CBAM helps the model focus on areas that are critical to benign and malignant classification, such as the irregular edges and invasive features of malignant lesions, through a combination of channel attention and spatial attention. This attention mechanism enables the model to effectively identify the key features of breast lesions in complex backgrounds, thereby reducing the misjudgment rate. In addition, the use of pre-trained weights speeds up the convergence of the model and improves the initial performance of the model under limited data conditions. The pre-trained weights provide RcdNet with a rich feature basis, enabling it to quickly adapt to the feature distribution of breast ultrasound images during fine-tuning, further enhancing the generalization performance of the model.

Breast cancer is a common malignant tumor in women. Early screening and diagnosis are crucial to improving the survival rate of patients. Traditional breast ultrasound diagnostic methods rely on the experience of physicians and may have certain subjectivity and diagnostic errors. RcdNet can effectively reduce manual misjudgment and improve the consistency and reliability of diagnosis through the automated feature extraction and precise classification of deep learning models, especially in the identification of early lesions. In addition, the high accuracy and interpretability of the RcdNet model provide technical support for clinical applications. Through visualization methods such as heat maps, physicians can clearly understand the basis of model decisions, which not only enhances the trust in the model prediction results, but also provides physicians with additional diagnostic reference information, which helps to more comprehensively evaluate the characteristics of lesions. This human-computer collaboration model can optimize the diagnostic process and improve diagnostic efficiency, especially in resource-limited medical environments, providing patients with better medical services. More importantly, the RcdNet model has good generalization ability and is suitable for different data sources and patient groups, showing strong potential for clinical promotion.

Although this study achieved good results in the classification of benign and malignant breast ultrasound images, there are still some limitations. First, the dataset used in this study is relatively limited. Although the model showed good generalization ability in both internal validation and external testing, it is still necessary to verify its performance on larger and more diverse datasets. Future studies should combine data from more sources, especially patient data of different races and age groups, to ensure the wide applicability of the model. Second, although RcdNet has excellent classification results on ultrasound images, in clinical practice, physicians usually combine multimodal images such as ultrasound, mammography, and magnetic resonance imaging (MRI) for comprehensive diagnosis. This study was based only on single ultrasound image data and failed to use multimodal information for auxiliary analysis, which may limit the diagnostic accuracy and comprehensiveness of the model to a certain extent. Future studies may consider introducing multimodal data fusion technology to improve the model’s ability to identify complex cases. Although heat map visualization provides support for the interpretability of the model, this technology still cannot fully reveal the internal decision logic of the deep learning model. Especially in the classification of highly complex lesions, physicians still need to be cautious in their reliance on the model. Further research could try more in-depth interpretative methods to improve the transparency of the model and make it more reliable and actionable in clinical applications.

## 5 Conclusion

This study proposed an innovative deep learning model, RcdNet, for benign and malignant classification of breast ultrasound images, and demonstrated excellent performance in internal validation and external testing. Compared with traditional models, RcdNet significantly improved the classification accuracy and generalization ability of the model through optimization strategies such as deep separable convolution and attention mechanism. At the same time, through the visualization analysis of heat maps, the model can provide intuitive interpretation of the lesion area, enhancing the clinical interpretability of the model. The results of the study show that RcdNet has important application potential in the early diagnosis and screening of breast cancer, and can be used as an auxiliary tool for physicians to improve the consistency and efficiency of diagnosis. In the future, through further data expansion and multimodal fusion, RcdNet is expected to be widely used in clinical practice and provide strong support for the precise diagnosis and treatment of breast cancer.

## Data Availability

The raw data supporting the conclusions of this article will be made available by the authors, without undue reservation.
